# Redefining autism in adulthood interventions *in* and *through* social virtual environments: an exploratory cross-cultural study

**DOI:** 10.3389/frcha.2026.1770399

**Published:** 2026-08-05

**Authors:** Vivian Darlene Grillo, Gaia Tanini, Federica Ciuciulla Rana, Paola Venuti

**Affiliations:** 1 Observation Diagnosis and Training Laboratory (ODFLab), Department of Psychology and Cognitive Sciences, University of Trento, Rovereto, TN, Italy; 2 Bruno Kessler Foundation (FBK), Trento, Trentino, Italia

**Keywords:** autism in adulthood, intervention design, neurodiversity-affirming care, social virtual environments, thematic analysis, virtual reality, cross-cultural study, low support needs

## Abstract

**Background:**

Autism intervention research has largely prioritized children and adolescents, with limited guidance for adults. Social virtual environments (SVEs; e.g., VRChat) are widely used by autistic adults for connection, yet their role as intervention settings remains under-examined. This study explores clinicians’ perspectives on feasibility, risks, and essential design requirements for clinician-informed SVE-based socio-relational interventions for autistic young adults with low support needs.

**Methods:**

We conducted nine semi-structured focus groups with Italian clinicians (*N* = 26) across three regions (Veneto, Trentino, Lazio) and five semi-structured international interviews (U.S. *n* = 4; Australia *n* = 1). Transcripts were analyzed via inductive, codebook-informed thematic analysis grounded in reflexive qualitative principles. Because the study involved four coders working across two linguistically distinct datasets, a shared coding framework was used as a pragmatic coordination tool to support transparency, interpretive consistency, and auditability across the research team (the intent was not to establish positivist inter-rater reliability or eliminate researcher subjectivity). Datasets were first coded separately and then integrated through matrix-based synthesis to identify contextually situated points of convergence and divergence.

**Results:**

Italian clinicians emphasized neurodiversity-affirming aims, context-dependent social difficulty (including anxiety/overwhelm), structured scaffolding and mediation, ecological generalization, and SVEs as conditional tools requiring boundaries. International clinicians foregrounded autonomy and choice, emotional safety in relation to masking, neurotype composition as an ingredient for belonging, identity-affirming collaboration, and regulation/repair practices. Across datasets, clinicians emphasized safety-contingent participation, non-negotiable personalization/controllability of demands (i.e., sensory, social), and the protective value of supported pauses and exits. Contextual differences centered on the dominant logic of support (competence-building scaffolding vs. liberation/identity integration), mediator-led structure vs. neurotype-aligned spaces, intersectionality as primary vs. contextual framing, and distinct risk emphases (avoidance/overuse vs. relational ambiguity and rejection).

**Conclusion:**

Clinicians conceptualize SVEs not as inherently therapeutic technologies but as conditional social infrastructures whose potential value depends on how safety, autonomy, and identity are supported. Clinician-informed SVE-based interventions for autistic adults may therefore require integrating structured mediation with explicit autonomy- and identity-affirming safeguards, offering a pathway beyond the traditional’ skills vs. acceptance’ divide in autism intervention research.

## 1 Introduction

Research on autism interventions has historically focused on children, and more recently on adolescents, leaving a persistent gap in understanding how evidence generated with these age groups translates into adulthood [e.g., (1)–(4)]. Autistic children and adolescents inevitably become adults, yet autistic adults have remained systematically underrepresented in both intervention research and clinical service development (5). This omission is reflected in a broader literature documenting pervasive challenges faced by autistic adults, including inadequate healthcare access and limited personalized support options [e.g., (6)].

A central dimension of these challenges concerns social participation. Autistic adults navigate a range of social environments — including workplaces, educational settings, and recreational spaces — that are predominantly structured around neurotypical norms and interaction styles [see: *Muted Group*; *Theory* (7)], placing highly variable demands on autistic individuals depending on context. For instance, research indicates that the need to mask [suppressing or concealing autistic traits in order to appear neurotypical — e.g., (8)] varies substantially across settings (9, 10). According to the literature, workplaces and formal educational contexts impose the most sustained masking demands [e.g., (11)]; in these settings, prolonged masking has been associated with burnout and exclusion from professional life, regardless of individual competence (10, 12). These outcomes are most likely also shaped by the nature and socio-relational or interpersonal dynamics of mixed-neurotype environments, as described by the *Double Empathy Problem* (13, 14). Recreational and community spaces present their own barriers, including sensory inaccessibility, implicit interactional expectations, and a prevailing emphasis on phatic exchange [social interaction aimed at maintaining relationships — see “socially based sociality” (15) — rather than conveying information], which sits in tension with the “interest-based sociality” (15) many autistic adults prefer [i.e., (9, 16); see: “Autistic Sociality”, (17, 18)]. Communication support needs further complicate participation: even autistic adults who use speech may experience intermittent or context-dependent communication difficulties that standard social environments do not accommodate (19). Overall, these context-specific demands — sensory, relational, and communicative — shape a landscape of social participation that is frequently taxing, inaccessible, or exclusionary for autistic adults [i.e., (20)].

Researchers have recently begun examining several technological avenues for supporting autistic adults’ social participation, though this landscape remains incompletely mapped. Social media platforms have received growing attention: autistic adults actively use them to build interest-based communities, build or maintain social connections, and express themselves “authentically” (9) in ways that offline settings rarely afford [e.g., (16)]. Digital platforms — like Reddit, Facebook groups, YouTube, and Twitter — offer affordances (including asynchronous communication, topic-structured interaction, and the ability to disengage without social penalty) that align with autistic social preferences (9, 11, 16, 21, 22). However, mainstream platforms are largely designed around neurotypical standards and expectations and, as Van Driel and colleagues (9) note, can create important psychosocial challenges. These challenges, the authors share, point to the need for social media platform developers to become more receptive and responsive to differences in socio-relational and communicative styles. Beyond social media, augmentative and alternative communication (AAC) tools have been explored as flexible supports across contexts, including for speaking adults whose communication needs vary by environment, content, and/or interlocutor — a population whose relationship with AAC has been significantly understudied relative to non-speaking autistic children (20). More broadly, Baillargeon and colleagues’ (10) recent systematic review of a decade of social computing research with neurodivergent participants found that this body of work remains medicalized [see: *Medical Model of Disability* (23)]: oriented predominantly toward children, toward normalization of neurodivergent behavior, and toward fitting autistic social practices to neurotypical standards instead of recognizing and supporting them on their own terms. Critically, another recent systematic review by Altin and collaborators (24) highlight that virtual reality (VR) technologies within social computing have typically been used in autism research as tools for social skill training and development. This orientation inevitably intersects with ongoing and highly contested debates surrounding interventions grounded in or relying on behavioral compliance [i.e., (25, 26)], rather than positioning VR as a space for social plurality and neurodivergent self-expression (24).

This historical trajectory matters for how the present study situates itself. VR-based interventions for autism have been developed for over three decades, yet they have primarily targeted children and adolescents, focused on skill acquisition directed toward neurotypical norms, and been designed with limited autistic input (27, 28). As awareness of these limitations has grown, scholars have increasingly advocated for a shift: instead of continuously developing new VR tools, there is growing recognition of the need to assess and adapt existing technologies for specific populations and purposes or goals (27, 28). SVEs — open-ended, user-populated three-dimensional platforms such as VRChat — represent an underexplored case in this regard. Unlike task-specific virtual environments designed for targeted behavioral training, SVEs are socially open spaces where interaction arises organically, making them structurally closer to Oldenberg's (29) notion of third places — informal spaces outside home and work where affiliation and identity can develop through low-stakes participation — than to clinical intervention tools. These platforms are already used widely by autistic adults for connection, community-building, and identity exploration (11), suggesting a naturally occurring alignment between SVE affordances and autistic social priorities. Yet this alignment has received no systematic examination from an intervention design standpoint.

In our previous work (11), we conducted an online netnography (30, 31) — using an adapted PRISMA framework [Preferred Reporting Items for Systematic Reviews and Meta-Analyses (32, 33)] — and applied a reflexive thematic analysis [e.g., (33, 34)] to investigate autistic adults’ use of digital environments and SVEs. Our findings indicate that autistic individuals actively engage in these platforms to socialize, advocate, and build peer communities. These results align with prior evidence that technology often represents a powerful interest and affinity for autistic people (35), and it may serve as a conduit for fostering neurodiverse communities (including allistic/neurotypical and autistic and/or other neurobiologies/neurotypes) within shared digital and virtual spaces. Despite this potential, SVEs remain unexplored as intervention settings for autistic adults. No study to date has examined how clinicians — the professionals centrally involved in designing and delivering such interventions — conceptualize the feasibility, risks, and design requirements of SVE-based support. This is the gap the present study addresses.

Thus, the contribution of the present study is not to speak for the end-users (autistic young adults) or to substitute professional perspectives for autistic lived experience. The contribution is to collect and examine how clinicians conceptualize the conditions under which social virtual environments could be used in neurodiversity-affirming, ethically responsible, and practically feasible ways. This focus is important because clinicians (psychologists, psychotherapists, and/or counselors) are often involved in designing, delivering, or adapting socio-relational supports, and are therefore positioned to identify implementation constraints, safeguarding needs, professional responsibilities, and tensions between traditional intervention models and neurodiversity-affirming practice. At the same time, clinicians’ perspectives remain only one layer of intervention design: they must be understood as complementary to, rather than replacing, autistic adults’ experiential knowledge and participatory involvement.This distinction is particularly relevant in the present study because the sample included different forms of professional standpoint. The Italian focus groups captured perspectives from clinicians working within more heterogeneous and, in some cases, traditionally structured clinical contexts, whereas the international interview sample consisted entirely of neurodivergent clinicians who publicly identify as autistic and/or ADHD prior to recruitment. These participants therefore contributed a hybrid form of expertise: professional clinical knowledge informed by lived neurodivergent standpoint. However, even where clinicians drew on lived experience, their contributions are analyzed here as clinician perspectives on support design and implementation, not as a substitute for autistic adults’ direct accounts of their own priorities, needs, and preferences. The present study should therefore be read as one step within a broader participatory research trajectory, in which clinician-informed design considerations require further integration with autistic-led and autistic adult perspectives. Generating professional perspectives rigorously and systematically is a necessary component to participatory co-design with autistic adults, which remains the essential next step of the more comprehensive research project this study is part of, and is therefore identified as a future direction of this research program. The study also captures perspectives across cultural and clinical contexts — including both traditionally oriented and neurodiversity-affirming clinical standpoints — recognizing that what clinicians understand “intervention” to mean varies and that this variation is itself analytically significant as it informs the objectives and delivery modalities of the intervention/support.

For autistic individuals across the lifespan, the broader stakes are substantial. Multifaceted challenges — including diagnostic complexity with co-occurring diagnoses (36), to stigma (37), polyvictimization [e.g., (38)], and pervasive epistemic injustice (39, 40) — converge on outcomes of isolation and loneliness. Research has identified disproportionately high rates of suicide risk within the autistic population (41), with loneliness identified as a particularly significant contributing factor linked to heightened risk [e.g., (42)]. Consequently, loneliness is increasingly considered and/or treated as a primary target for intervention development [e.g., (43)]. SVEs, designed appropriately, may represent one pathway toward reducing isolation and supporting belonging — not as a replacement for in-person social life, but as a potentially regulated and personalized intermediate ecology for engagement and connection, particularly when conventional settings are inaccessible or excessively demanding.

Situated between traditional in-person interaction programs [i.e., PEERS; e.g., (44)] and technologically advanced interventions using virtual agents [e.g., (45)–(47)], SVEs offer a distinct set of affordances: user-driven interaction, customizable avatars, variable sensory environments, and the possibility of neurotype-aligned communities. These platformsappear to create potential conditions for bridging the Double Empathy Problem (13, 14) between autistic and non-autistic individuals in contexts of curiosity and mutual understanding (11); although whether and how these affordances can be harnessed within a structured intervention remains an open empirical and clinical question (11).

### 1.1 Objectives

This exploratory study aimed to gather and analyze clinician-informed perspectives from licensed clinicians on the feasibility, design requirements, ethical risks, and implementation conditions of SVE-based socio-relational interventions for autistic young adults [L1, low support needs; 18 – 40 years; DSM-5-TR, (48)]. Specifically, from the professionals’ perspective, the study sought to:

1. Identify core clinician-informed design characteristics for socio-relational interventions in SVEs, drawing on established practices from in-real-life social interventions and prior virtual reality-based approaches with autistic individuals.
2. Examine perceived opportunities and limits of using SVEs platforms, such as VRChat, for supporting connection, belonging, social participation, and reduced isolation among autistic your adults.
3. Assess potential risks, limitations, and ethical considerations related to deploying interventions in virtual social contexts, with a focus on safety, accessibility, autonomy, and unintended consequences.
4. Integrate professional expertise into intervention design by highlighting clinicians’ recommendations for adapting, personalizing, and responsibly implementing digital and virtual tools to support autistic adults.
5. Ensure intervention cultural responsiveness by considering how clinician reasoning may vary across contextually situated professional, institutional, and advocacy environments, without treating the samples as representative national synthesis groups. Thus, potentially enhancing intervention accessibility and relevance across different autistic populations.

## 2 Materials and methods

### 2.1 Research design: methods

This study employed a qualitative, exploratory design using semi-structured focus groups (Italian sample) and semi-structured in-depth individual interviews (international sample) to examine clinicians’ perspectives and professional reasoning about interventions supporting autistic young adults. Both formats were developed around the same six core thematic areas and guided by the same iteratively refined topic guide (see Section 2.3.), allowing for comparability across methods while preserving the flexibility necessary for participant-led discussion or sharing.

The choice of focus groups for the Italian sample stemmed from its collaborative, dialogic format that was expected to generate richer, emergent data through professional exchange, debate, and shared sense-making among clinicians working in similar national service contexts.

Individual interviews were selected for the international sample (American and Australian clinicians) to maximize depth and candor given the smaller, geographically dispersed group, and because the exploratory extension of the design did not permit group coordination.

The study received ethical approval (protocol code: Progetto Grillo; CE registry number: A978) by the Ethics Committee of the Autonomous Province of Trento's local health authority (Azienda Provinciale per i Servizi Sanitari di Trento, APSS), and all participants provided informed consent in person or online prior to data collection.

### 2.2 Sampling strategy: heterogeneity as an analytic resource

Qualitative research, by its very nature, affords methodological flexibility and foregrounds reflexivity as a core practice of inquiry. Within the scope of the broader research project of which this study is part, we critically examined our sampling strategy and recognized that the autistic population under investigation is embedded in diverse cultural contexts and countries of origin. Relying solely on Italian professionals risked limiting the cultural sensitivity and transferability of the insights generated, particularly in relation to designing interventions intended to be inclusive, adaptable, and contextually responsive.

Previous literature also suggests that neurodiversity discourse and autism advocacy have developed differently across national contexts, with Anglophone countries generally showing a longer and more explicit engagement with neurodiversity paradigms than Italy, where these debates have emerged more recently and remain comparatively less institutionalized [e.g., (49)–(52)].

In addition, the institutional organization of autism-related support differs across contexts: in Italy, services are shaped by a regionalized public health system and by uneven adult autism pathways, whereas U.S.A. and Australian contexts include more visible neurodiversity-affirming private-practice and community-advocacy spaces, alongside access barriers linked to insurance, cost, geography, and service availability. These differences do not make the present study a representative national synthesis; instead, they provide a rationale for treating contextual variation as an interpretive resource for understanding how clinicians reason about neurodiversity-affirming SVE-based interventions. In other words, including clinicians from the United States and Australia enabled the exploration of how different socio-cultural and professional contexts may shape understandings of neurodivergence and intervention practices, thereby informing more culturally sensitive design perspectives. Accordingly, the sample was extended to include professionals from different countries in order to enrich the interpretive depth of the analysis and to situate clinicians’ reasoning within different professional, institutional, and advocacy contexts, rather than to establish generalizable cross-national claims.

Therefore, participant recruitment prioritized professional and contextual heterogeneity as a means of enriching the data rather than achieving statistical representativeness. Clinicians were eligible to participate if they held an active license in psychology, psychotherapy, and/or counselling and had documented clinical experience working with autistic young adults (18 – 40 years, Level 1/low support needs) across in-person and/or virtual reality-based interventions. Sampling continued until thematic sufficiency was reached within each dataset.

The two samples were recruited through strategically distinct channels, reflecting different and complementary forms of clinical expertise. The Italian sample was recruited through nationally recognized, regionally located autism and ex-Asperger's clinical associations across Veneto, Trentino, and Lazio, supplemented by snowball referrals. As clinicians’ theoretical orientations could not be ascertained prior to recruitment, this approach yielded a heterogeneous sample spanning both traditional (medical) and more neurodiversity-affirming clinical perspectives, as well as both neurotypical and neurodivergent practitioners (who were not asked to self-disclose). This heterogeneity is treated as an analytic resource rather than a limitation. The international sample was recruited through a purposive strategy designed to access clinicians with both lived and professional experience of autism: four of the five participants were identified via Instagram, where they publicly identified as neurodivergent (autistic and/or ADHD with/without autistic children of their own); the fifth was recruited via word of mouth by one of the previous four. These distinct recruitment strategies reflect the study's interest in capturing complementary epistemic standpoints — institutionally embedded clinical expertise on one hand, and insider, lived-experience-informed clinical expertise on the other. Accordingly, neither sample is intended to be representative of all clinical practice in its respective country or worldwide; both samples are purposely constituted to illuminate specific perspectives relevant to the research interest of this study: *what is being done, effectively, in interventions for autistic young adults?* Thus, the neurotype composition of the two samples differed systematically: the Italian sample was mixed (neurotypical and neurodivergent practitioners), while the international sample was entirely neurodivergent (autistic and/or ADHD). This difference is not treated as a confound to be controlled, but as a source of richer data, characterized by multiple epistemic standpoints, that can help us depict a better understanding of what is currently being done by the clinician population.

#### 2.2.1 Italian sample

Nine semi-structured focus groups were conducted with *N* = 26 Italian clinicians (psychologists, psychotherapists, and/or counselors) working with autistic young adults across three regions: Veneto, Trentino, and Lazio. Participants’ ages ranged from 30 to 58 years (*M* = 10; *F* = 16), with clinical experience ranging from 3 to 25 years. The sample was mixed, thus including neurotypical and neurodivergent practitioners. The proportion of neurodivergent clinicians within the Italian sample is reported descriptively but was not used as a stratifying variable in analysis; its potential influence on the thematic results is discussed where relevant (see “*Clinicians’ neurotype: as an inherent bias or as an interpretive point of view?*” in the “Discussion section”).

Participants were distributed into focus groups of three, with the exception of one group of two (one participant was unable to attend due to unexpected illness). Each session lasted approximately one hour. Four sessions were conducted and recorded online via Zoom to enable simultaneous data collection across geographically dispersed regions; five sessions were conducted in person and audio-recorded using a physical recording device.

#### 2.2.2 International sample

This sample included five individual, in-depth, semi-structured interviews conducted with Italian-American English-speaking clinicians from the United States (*N* = 4) and Australia (*N* = 1). All participants identified as neurodivergent and are publicly known within autistic-led professional communities that work with autistic people. All interviews were conducted via Zoom following online informed consent and lasted approximately one hour each. Thematic sufficiency (or saturation) was reached for the analytic purpose of developing a contextually situated account of this sample's perspectives and later considering how these perspectives resonated with, extended, or differed from the Italian dataset.

This sample was not designed to represent English-speaking clinical practice broadly, nor to achieve parity with the Italian sample in size (especially given the differentiation in methods). Instead, it was purposively constituted to access a form of expertise that the Italian sample could not provide: clinical knowledge grounded simultaneously in professional training and autistic lived experience abroad. Findings from this sample are therefore presented as contextually situated perspectives whose value lies in conceptual depth and in the nuances they add to the broader analysis, not in generalizability.

We emphasize that the international sample is not intended to be representative of U.S. or Australian clinical practice more broadly: its small size, its exclusively neurodivergent composition, and its recruitment criteria, are used and presented within this framework as contextually situated perspectives, used to identify conceptually meaningful convergences and divergences rather than to make population-level claims. We invite the reader to interpret the cross-contextual synthesis with this caveat in mind. Thus, the exclusively neurodivergent profile of this sample may have amplified attention to concepts close to neurodiversity-affirming views [i.e., identity affirmation, un-camouflaging: masking and compensating, intersectionality, and political ethics of care; e.g., (53)] — perspectives that are analytically valuable and may not have otherwise transpired through the Italian sample alone. All standpoints were considered during the analysis and addressed in the following sections in order to best uncover current epistemologies in intervention ideation, design and delivery.

### 2.3 Materials development and data collection

Focus group and interview guides were developed through an initial desk research phase drawing on existing (and lack of) literature on adult autism interventions, social skills programs, virtual reality-based approaches, and autistic adults’ use of digital environments. The resulting semi-structured topic guide was organized around six core thematic areas:

1. Effective approaches to socio-relational support
2. Tailoring environments to individual sensory and social needs
3. Familiarity with and orientation/comfort with and within virtual environments
4. Identifying elements of a sensory-friendly virtual space
5. Potential challenges and solutions for intervention implementation
6. Feedback on initial SVE (Social Virtual Environment) intervention design concepts

The guide was applied consistently across both focus groups and individual interviews to support comparability between methods, while remaining flexible and responsive to the natural development of participants’ exchanges. Following established practice in qualitative inquiry, the guide was iteratively refined across data collection phases in response to emerging insights and/or data saturation within the investigation of certain avenues. No substantive thematic content was added or removed between the two formats and/or between sessions. Overall, focus groups and interviews followed an identical topic structure, differing only in format and language, which was adapted to reflect the conversational register of the participant(s). All focus groups and interviews were audio-video recorded with participants’ prior consent. Recordings were stored on the University of Trento's institutional secure server, managed and maintained by the IT infrastructure of the ODFLab (Rovereto), ensuring institutional-level data protection and access control. Recordings were manually transcribed in full by Italian- and Italian-American English-speaking members of the research team. Data were anonymized prior to analysis: all identifying information (including names, locations, and any contextually identifying details) was systematically replaced with bracketed placeholders [e.g., [SENSITIVE DATA], [ANONYMIZED DATA]], rendering participants entirely non-identifiable at the transcript level. Anonymized transcripts and coded data were stored and managed separately from the original recordings, in institutionally encrypted, double password-protected Google Drive folders with access restricted to named coders and members of the research team.

### 2.4 Analytic approach

All data were analyzed using an inductive (54) thematic analysis approach (55, 56) grounded in the reflexive tradition established by Braun and Clarke [Reflexive Thematic Analysis, or RTA; (57)], which treats theme development as an active, iterative, and interpretive process shaped by the researchers’ analytic positioning (not by a procedure of neutral extraction). This approach was considered appropriate for the study's aim — understanding how clinicians conceptualize, reason about, and give meaning to SVE-based support — which produces latent, interpretive themes rather than surface-level descriptive ones. Reflexivity was sustained throughout analysis via memo-writing, peer-debriefing, and audit trail documentation (described below). The analysis was facilitated by NVivo (QSR International) in order to systematically generate situated and interpretive themes. However, the specific structural features of this study (four independent coders working across two linguistically distinct datasets) required procedural adaptations that draw on codebook thematic analysis principles (33, 34, 58) to manage cross-coder consistency and ensure transparent, auditable decisions. Codebook procedures were therefore incorporated as a practical scaffold for sustaining rigor within a reflexive analytic framework: one that preserves the interpretive depth of RTA while making the reasoning behind thematic decisions traceable and verifiable across coders and datasets. Moreover, having used a macro themed-based guide across samples at the time of data collection to allow comparability, the inclusion of the codebook approach was inevitable. This integration (mixed methods) is consistent with approaches adopted in comparable multi-coder qualitative studies and literature describing the reasoning, and advantages, behind choosing a mixed approach [e.g., (59)–(61)].

The Italian and international transcripts were first coded independently and in parallel: the nine Italian transcripts were analyzed by two native Italian-speaking coders, and the five English transcripts were analyzed by two native Italian-American English speaking coders. To strengthen rigor and trustworthiness (62, 63), within each pair, coders worked independently before meeting to reconcile discrepancies through iterative discussion, treating disagreements as analytic opportunities to surface interpretive assumptions. Unresolved differences were escalated to the full research team, which included licensed clinicians with relevant expertise, ensuring that thematic decisions reflected collective interpretive judgement via consensus-building.

To integrate the two datasets, we adopted a matrix-based synthesis procedure. This process was used to identify points of resonance, variation, and contextual nuance across the Italian and international datasets. Findings were further consolidated through data triangulation, whereby insights from one sample were used to validate, challenge, or expand those from the other, ensuring that the resulting framework reflected both trustworthiness and cultural responsiveness.

Throughout analysis, an audit trail was maintained documenting coding decisions, codebook revisions, and theme development stages, thereby ensuring transparency and addressing the criteria of dependability and confirmability (64). Regular peer debriefing sessions (64) were held within the research team, broadening interpretive depth and mitigating individual researcher bias, thus contributing to analytical rigor and investigator triangulation, while theoretical triangulation was ensured through the integration of perspectives from autism research, clinical psychology, and digital/virtual intervention design. To enhance credibility and transferability, we engaged in reflexive memo-writing throughout analysis.

We note that no formal analytic procedure was implemented to systematically separate the thematic contributions of neurodivergent vs. neurotypical clinicians within the Italian dataset. While neurotype composition is discussed interpretively in the Discussion, the data collected (see Section 2.2) does not permit systematic attribution of specific themes or emphases to clinician neurotype. Future research employing stratified sampling by neurotype would allow for more rigorous examination of this variable, which falls outside the scope of this specific study.

## 3 Results

### 3.1 Italian clinicians’ perspectives on interventions for autistic young adults

Analysis of nine focus groups with Italian clinicians revealed a coherent set of themes describing how professionals conceptualize support for autistic young adults and the conditions under which socio-relational interventions may be effective. While participants varied in professional background and setting, their accounts showed substantial convergence around the aims of intervention, the nature of social difficulties, and the role of structured and mediated environments. The findings are organized into five themes.

#### 3.1.1 Theme 1: reframing the aims of intervention through a neurodiversity-affirming lens

Across all focus groups, clinicians articulated a clear shift away from intervention models aimed at normalizing autistic behaviors through compliance. Instead, participants emphasized wellbeing, functional adaptation, and personal agency as primary goals of support. Intervention was not framed as a process of correcting differences, but as providing tools that allow individuals to navigate social environments while remaining authentic to their neurodivergent identity.

Several clinicians explicitly contrasted earlier deficit-based paradigms with a neurodiversity-affirming perspective, noting that the purpose of therapeutic work was not to reduce visible signs of autism but to support quality of life and self-understanding. One participant stated:

“It's not that I have to teach you to adapt to my world so that you're less different and it's less visible that you're autistic. The paradigm completely changes with the concept of neurodiversity.” (FG2)

Intervention goals were thus described as collaborative and person-centered, focusing on helping individuals feel well, develop strategies, and understand their own functioning rather than conform to external social expectations. As another clinician explained:

“Our therapeutic work is not aimed at changing anyone, but rather at providing strategies that allow for functioning and wellbeing.” (FG4)

This reframing also involved challenging broader social assumptions, with clinicians highlighting the need for inclusion to be understood as responsiveness to diverse needs rather than the elimination of difference.

#### 3.1.2 Theme 2: social difficulties as relational, emotional, and context-dependent

Clinicians consistently described social difficulties in autistic adults as complex and multifaceted, emerging from the interaction between individual processing styles, emotional states, and social contexts. Rather than attributing challenges to a lack of social motivation, participants emphasized anxiety, uncertainty, and difficulties interpreting implicit social norms.

Many clinicians highlighted difficulties in decoding social situations, particularly in group contexts, where multiple cues and rapid exchanges increase cognitive and emotional load. One participant observed:

“There is often a difficulty in decoding social situations, with misunderstandings and errors in reading the context. This becomes even more complicated in larger groups.” (FG5)

Social anxiety was repeatedly identified as a key factor that limits initiative and reinforces avoidance. Clinicians noted that uncertainty about how one is perceived by others often leads individuals to remain on the defensive, reducing opportunities for interaction. As one clinician described:

“There is an anxiety that keeps them quite defensive, protected, and often avoidant.” (FG5)

In addition, participants discussed relational fatigue and overload, explaining that sustained social interaction can be physically and emotionally exhausting. The need for pauses and recovery time was framed as a legitimate aspect of autistic social experience rather than a lack of engagement:

“There is an overload in managing the relationship, which then leads to tiredness. Some people need moments of pause to recover before they can return to interacting.” (FG5)

Together, these accounts position social difficulties as relational and situational phenomena, shaped by both internal states and external demands.

#### 3.1.3 Theme 3: the necessity of scaffolding (structure, mediation, and graduality)

A central theme across focus groups was the importance of scaffolding in socio-relational interventions. Clinicians emphasized that predictability, clear structure, and professional mediation are not constraints on autonomy but enabling conditions for participation.

Participants described structured support as essential for reducing anxiety and making social environments more manageable. This included anticipating activities, clarifying expectations, and breaking interactions into sequential steps. One clinician noted:

“Structure and anticipation are indispensable. The environment needs to be predictable to reduce anxiety related to the unexpected.” (FG3)

In addition to verbal scaffolding, clinicians highlighted the value of sustaining augmentative and alternative communication (AAC) supports beyond childhood, describing AAC as a practical tool for reducing cognitive load by externalizing expectations into clear, visual formats. Participants noted that these supports are often implemented in childhood services but become less available in adulthood, despite remaining clinically useful for autistic adults. They recommend integrating AAC into both in-person and SVE delivery through structured visual agendas, step-by-step sequences, symbol-supported “social stories”, and embedded prompts that clarify procedures and interactional norms. This was presented as an inclusion strategy that supports comprehension, planning, and self-advocacy — particularly for individuals with literacy difficulties or high executive demands (FG9).

Professional mediation was also described as crucial, particularly in group contexts. Clinicians portrayed themselves as facilitators who help initiate interaction, regulate dynamics, and support reflective processing, rather than as directors of behavior. As one participant explained:

“We position ourselves as catalysts, giving a push so that things can happen, while carefully controlling the variables.” (FG6)

Gradual progression — from individual work to small groups and, eventually, broader social contexts — was repeatedly emphasized. Clinicians cautioned against premature exposure to complex situations, stressing the interdependence of individual and group work in supporting generalization.

#### 3.1.4 Theme 4: ecological and mediated contexts as preconditions for adult support

Participants expressed skepticism toward interventions confined to traditional clinical settings, particularly for autistic adults. While acknowledging the usefulness of structured training in some cases, clinicians argued that overly artificial contexts fail to capture the unpredictability and emotional complexity of everyday social life.

Ecological and semi-naturalistic settings were described as more effective for supporting meaningful learning and transfer. One clinician remarked:

“Working only in the studio is very limiting. In real contexts, variables emerge that you simply can't reproduce in a protected setting.” (FG7)

Several participants described interventions embedded in informal activities, community spaces, or shared projects, where social interaction arises organically rather than being imposed as an explicit task. These contexts were seen as facilitating motivation and authenticity, while still allowing for professional support and reflection.

Importantly, clinicians emphasized that ecological does not mean unstructured. Even in informal settings, careful preparation and mediation were described as necessary to ensure safety and coherence.

#### 3.1.5 Theme 5: virtual environments as regulated social spaces (opportunities and tensions)

When discussing virtual environments, clinicians adopted a nuanced and cautious stance. Virtual spaces were described as potentially supportive contexts that can reduce certain barriers to social interaction, particularly anxiety associated with face-to-face encounters. Professionals shared that the use of avatars and mediated interaction in virtual reality facilitate engagement for some individuals by lowering social pressure and increasing spontaneity (“Interacting through an avatar can reduce anxiety, allowing for more spontaneity in the interaction.” FG4).

Clinicians also discussed the delivery modality as a practical determinant of accessibility and retention, distinguishing between headset-based VR and desktop-based participation. Several participants suggested that desktop access may be less fatiguing (i.e., motion-sickness) and more familiar for some users, thereby minimizing barriers unrelated to the intervention's socio-relational goals. Modality was also framed as an equity and feasibility issue because headsets are not universally available or affordable, clinicians recommended designing the intervention so it can be delivered remotely via desktop when needed. Some proposed an initial onboarding phase (ideally with at least one in-person meeting) to introduce the platform, allow participants to try the technology where possible, and align expectations before transitioning to remote sessions (FG5).

At the same time, participants emphasized that virtual environments are not universally suitable and may pose risks if used without appropriate regulation. Concerns included the possibility of increased isolation, difficulties distinguishing between virtual and offline relationships, and over-reliance on digital interaction.

Clinicians stressed that the value of virtual environments depends on individual characteristics, professional mediation, and integration with offline experiences. As one participant summarized:

“It can be a powerful tool, but it's a double-edged sword. Without guidance, there's the risk that everything remains confined to that (virtual) context.” (FG3)

Overall, virtual environments were framed as conditional spaces whose effectiveness relies on structure, monitoring, and alignments with broader support goals.

### 3.2 International (American/Australian) clinicians: thematic findings

While the international sample was comparatively smaller than the Italian dataset and should therefore be interpreted cautiously, the interviews nonetheless offered analytically rich insights into how a group of American and Australian clinicians conceptualized support for autistic young adults. Rather than being treated as representative of their broader national clinical cultures, these findings are presented as exploratory perspectives to be compared and integrated with the Italian sample's.

Across the interviews, participants consistently framed supportive practice as identity-affirming, autonomy-preserving, and context-aware. Instead of positioning intervention as skill acquisition toward neurotypical norms, clinicians described support as helping autistic individuals: (i) “understand social environments as systems” organized around particular norms and expectations (predominantly neurotypical), (ii) develop informed choice about participation in those norms, and (iii) build access to relationships and spaces where authenticity is safer and more sustainable.

#### 3.2.1 Theme 1: autonomy and choice as the organizing principles of “support”

International clinicians frequently framed adult support through the lens of choice, emphasizing that adult autistic people benefit from transparent information about social conventions without an obligation to comply. One clinician (Interview 5) described the clinical task as layering “neurotypical norms” as optional contextual knowledge and explicitly reinforcing personal choice about whether engaging those norms is “worth it” in a given situation. Support is contextualized as helping autistic people develop agency — including the ability to evaluate contexts (e.g., school, parties, workplace norms) and decide what level of adaptation is worth the cost.

This emphasis on choice was also linked to structural constraints and privilege (e.g., socioeconomic status, race, gender identity, communication profile), with clinicians noting that “choice” is unevenly distributed, and that intervention design must be inclusive and autistic-centered to avoid reproducing inequities.

#### 3.2.2 Theme 2: emotional safety, masking, and the pragmatics of authenticity

A second central theme concerned the relationship between emotional safety and masking. Clinicians explicitly rejected the assumption that “unmasking” is universally safe or feasible, instead treating it as an adaptive strategy that may be necessary in specific settings (e.g., employment, high-stimulation public environments) and a protective “part” (referencing Internal Family Systems, or Parts Work) that can be appreciated rather than pathologized. Authenticity is described as an outcome that requires conditions of safety rather than a demand placed on the autistic individual. One clinician stated that for many autistic people it is “not safe or possible to unmask all the time” (Interview 5), including in professional contexts where power and control are comparatively high, highlighting how “being perceived” in mixed-neurotype spaces can evoke automatic masking.

In this framing, support involves helping clients identify where authenticity is realistic, where strategic masking may be necessary, and how to recover a sense of self when long-term masking has become habitual and non-conscious.

#### 3.2.3 Theme 3: autistic-centered spaces and neurotype composition as a discrimination tool for belonging

Clinicians described neurotype composition (who is in the social space) as an active ingredient that can determine whether a social environment becomes emotionally safe. *Who is in the space* matters: mixed-neurotype environments may remain difficult even when they are nominally affirming, because autistic people may automatically mask in response to surveillance-like social evaluation (“the feeling of being perceived”). Clinicians suggest that predominantly autistic or broadly neurodivergent spaces may reduce the pressure to perform neurotypical social norms and thereby support comfort and increase ease of connection, some explicitly linking this to the “Double Empathy Problem” framing to explain why shared neurotype can facilitate mutual understanding.

This theme positioned social virtual environments as potentially valuable not because they can teach normative social skills, but because they can support access to “people you can connect to” and a sense of third-place belonging (Interview 4, USA).

#### 3.2.4 Theme 4: identity-affirming practice as cultural stance and clinical ethics: collaboration over hierarchy (“radical genuineness”)

Another theme concerned the clinicians’ relational position or role within the therapeutic dynamic: participants described therapeutic relationships as more effective when they resist “expert/fixing” hierarchies and instead model collaborative curiosity, creating conditions where clients can correct misunderstandings and advocate for needs. Thus, international clinicians treated language and identity-affirming practice as part of the intervention context rather than an optional add-on. Participants described systematically asking clients what language model they prefer (i.e., identity-first vs. person-first), then reflecting that preference consistently in interaction and advocacy materials.

Identity was also discussed as explicitly political and intersectional (e.g., neurodivergence, queerness, gender diversity), with clinicians describing autistic-affirming work as inseparable from broader commitments to accessibility and justice.

#### 3.2.5 Theme 5: regulation and relational repair as core implementation needs

Several clinicians described regulation and co-regulation as prerequisites for social participation, providing concrete examples of grounding supports that reduce the cognitive effort required for self-regulation. In particular, repetitive motor/physical movements and/or stimming was framed as an inherently regulating and identity-affirming practice, rather than as a behavior to be suppressed.

Clinicians also highlighted the importance of relational repair practices, including explicit scaffolding for how to recover after misunderstandings or dysregulation, particularly in contexts where sensory stress and social ambiguity may interact.

### 3.3 Cross-cultural synthesis: points of convergence and divergence

The following section presents primary convergences and divergences between Italian and American/Australian professionals’ perspectives and approaches. This synthesis is not intended as a formal comparative analysis between national groups; rather, it uses the two datasets to identify contextually situated patterns in clinicians’ reasoning. Given the exploratory and unevenly sized samples, differences are interpreted as situated emphases instead of population-level contrasts.

#### 3.3.1 Convergences

Despite differences in conceptual details, Italian and International clinicians converged on several key principles relevant to In-Real-Life and SVE-based support or intervention design.

##### 3.3.1.1 Convergence 1: social participation is safety-contingent and requires predictability, pacing, and preparatory scaffolding

Across both datasets, clinicians framed social participation as *unsafe by default* unless uncertainty, overload, and exposure are actively managed. In other words, social participation is contingent on individual profiles and context-fit, rather than a one-size-fits-all protocol. Italian clinicians repeatedly emphasized sensory control, structured preparation and gradual, paced entry into social contexts, including virtual ones, to prevent frustration and withdrawal:

“The initial phase needs to be sufficiently structured… the risk of giving too much freedom is that nothing ends up happening… at the beginning, everyone requires some degree of facilitation/prompting” (FG5)

This logic of preparatory containment was echoed in all Italian groups, where clinicians stressed the importance of small groups and staged expansion:

“I would start with small groups, as starting in smaller group settings is particularly important for them” (FG6)

International clinicians converged on the same principle, albeit framed in terms of emotional safety and expectation management, particularly in online contexts:

“Being prepared is kind of key… helping them understand what to expect gives context so it's not just them” (Interview 4, USA)

Within interventions, both groups of clinicians described the need for support structures that reduce demand and uncertainty, such as preparation, moderation, and stepwise engagement. International clinicians framed these structures primarily as a way to enable autonomy and safe authenticity, for example by creating an explicitly open and affirming environment modeled by facilitators. While Italian clinicians focused more on *structural pacing* (e.g., speed of progression; group size; degree of freedom), international clinicians placed greater emphasis on *cognitive-emotional preparedness* (anticipating rejection, ambiguity, or relational rupture). These approaches represent complementary rather than conflicting safety mechanisms, and both groups claim self-awareness and self-understanding are essential for better intervention outcomes and improved quality of life. Furthermore, both samples highly recommend teaching assertive communication to autistic people to navigate social interactions and environments safely and constructively.

##### 3.3.1.2 Convergence 2: virtual environments can facilitate access to connection, belonging and authentic self-expression (central outcomes) but must be aware of potential isolation or relational harm

Italian clinicians consistently described SVEs as potentially useful for individuals who otherwise isolate, particularly as a “gym” for desensitization and initial engagement:

“It could be useful as a kind of training ground… but one that is clearly bounded, with a defined beginning and end” (FG7)

However, they simultaneously warned against replacement of offline life, explicitly naming the risk of “Covid effect”:

“The risk is that it could become a form of avoidance of social situations that are hard to manage” (FG7)

International clinicians converged on the ambivalence of online spaces, describing them as lower-risk entry points that nevertheless expose autistic adults to relational fragility, such as ghosting:

“Online space tend to be really good; the negative side is that is can be easy for those connections to ghost them, and those feelings of suicidal ideation go up” (Interview 4, USA)

Italian clinicians primarily framed risk as *avoidant drift* (staying in the virtual; online isolation), whereas international clinicians foregrounded *interpersonal rupture* (being abandoned, dismissed or misled). Together, these point to different but equally salient failure modes of online sociality that need to be kept in mind during intervention implementation.

Across contexts, clinicians conceptualized value in SVEs primarily as the potential to support connection, community finding and sense of belonging rather than to learn better compliance-strategies to perform normative social behavior, aligning with the “third place” framing referenced in the interviews.

##### 3.3.1.3 Convergence 3: personalization and controllability of sensory and interactional demands are non-negotiable for inclusivity

Italian focus groups repeatedly emphasized the need to actively manage sensory load and interactional complexity, framing personalization as an inclusion strategy rather than an accommodation for a (neuro-)minority:

“Being attentive and attending to specific vulnerabilities constitutes a set of precautions that can support more inclusive forms of change” (FG2)

Similarly, international clinicians normalized regulation supports and autistic modes of interaction as foundational engagement:

“Autistic connection looks different giving them a way to interact with each other, starting from what lights them up” (Interview 1, USA)

Italian clinicians talk about environmental manipulation (stimuli, group size), while international clinicians refer to interest-based engagement and neurotype-affirming interaction styles. Both converge on reducing demand rather than increasing tolerance. Overall, controllability of sensory and interactional demands are non-negotiable.

##### 3.3.1.4 Convergence 4: a supported exit, pause, or decompression option is protective rather than avoidant

Italian clinicians explicitly proposed built-in escape or pause options within interventions to prevent overload:

“I ask for more breaks… I can then experience, in a socially appropriate way, a situation that is otherwise a source of frustration for me” (FG7; clinician is referring to the “taking breaks strategy” used with autistic people to support self-regulation. The clinician is adopting the patient's first-person voice, illustrating an internalized behavioral script in clinical practice)

International clinicians framed similar strategies as protective preparation, particularly in emotionally high-stakes online interactions:

“[…] preparation for rejection, preparation for how to manage the direction of relationships […]” (Interview 4, USA)

Both datasets frame withdrawal as instrumental and necessary, not as failure. Disengagement is conceptualized as self-regulation when embedded in a structured, reflective process.

#### 3.3.2 Divergences

A preliminary and immediately visible divergence concerned clinical language. Italian clinicians referred to the autistic people as “patients”, while American and Australian professionals used “clients”.

Beyond terminology, the most salient divergence concerned *how* clinicians conceptualized autism support, which is articulated throughout the following paragraphs.

##### 3.3.2.1 Divergence 1: core aim of support: “structured, competence-building adaptation” vs. “liberation: agency and identity integration”

The Italian focus groups emphasized structure, progressive adaptation with relational scaffolding and skill generalization. They consistently oriented intervention toward guided adaptation that requires skill rehearsal, decoding, and gradual competence-building:

“[…] working to acquire or develop tools for managing an initial form/early stage of social interaction, one that is somewhat less forced” (FG6)

In contrast, international clinicians centered “agency”, “autonomy”, “identity affirmation”, and “choice” as the primary therapeutic logic of adult support, explicitly resisting prescriptive models of change:

“Helping them (autistic clients) understand that masking (or re-enacting “neurotipicality”) is a part (of who they are), not something bad, authenticity depends on safety.” (Interview 5, Australia)

This divergence does not reflect disagreement about ethics, but rather different priorities or primary levers: Italian clinicians focus on *how to help people function*, international clinicians on *how to help people choose when and whether to function* within dominant norms.

##### 3.3.2.2 Divergence 2: normative social knowledge as optional context vs. training target

International clinicians described offering explanations of neurotypical norms as context that supports decision-making (*“you get to decide how you wanna take it”*), explicitly resisting “*teaching compliance*”. This differed in emphasis from the Italian accounts centered on mentalization, decoding social situations, and structured skills practice.

This divergence can be understood in light of an ongoing debate within the autism intervention literature. Structured social skills training programs — such as PEERS (65), ABA and TEACCH practices and interventions [e.g., (66)], and Theory of Mind-based curricula [e.g., (67)] — have a strong evidence base in terms of knowledge acquisition and observer-rated social competence, and remain influential in international clinical contexts. However, a growing body of research questions whether compliance-oriented social skills training improves autistic wellbeing or, conversely, increases masking and associated mental health costs [e.g., (53)]. Autistic self-advocates and participatory researchers have raised concerns that such programs implicitly target neurotypical conformity as the endpoint, without adequate attention to autistic adults' own relational goals or values [e.g., (68)]. The international clinicians’ position — offering normative knowledge as optional context rather than an intervention target — reflects this critical strand of the literature and is consistent with a shift toward self-determination as an organizing framework for adult support (69, 70). The Italian clinicians’ position, by contrast, is consistent with a graduated model of social participation in which structured skill-building is understood as scaffolding toward greater autonomy rather than as compliance *per se*, aligning with established models of supported participation in European and international developmental psychotherapy traditions. Overall, these positions may represent different entry points into what appears to be a shared goal: both seek to expand autistic individuals’ capacity to navigate social environments on their own terms, but differ in whether normative social knowledge is treated as a means to that end or a potential constraint on it.

##### 3.3.2.3 Divergence 3: professional mediation and structure vs. neurotype composition

Italian clinicians debated the balance between promoting autonomy and autodetermination, and ensuring professional containment, consistently emphasizing the psychologist/therapist's role as an active mediator within both in-person and virtual contexts. In this framing, the presence of a professional within the social environment — particularly in VR — was described as a necessary condition to prevent abandonment, overload, or unstructured exposure.

Italian clinicians described the mediator as responsible for actively managing and supporting interaction, both between autistic participants and within the environment itself. Although autistic adults were described as generally motivated to socialize, clinicians noted that many experience difficulties with social initiation; professional mediation was therefore seen as essential to facilitate entry into interaction and sustain engagement.

In addition, Italian clinicians emphasized the mediator's role in environmental control and anticipation. This included monitoring and adjusting sensory stimuli in response to individual hypersensitivities, managing environmental unpredictability, and structuring activities in advance to create coherent and measured expectations aligned with each participant's profile. Through this regulation, clinicians aimed to reduce anxiety and prevent escalation or withdrawal.

A further function attributed to the mediator concerned the promotion of self-awareness and self-monitoring (which also appeared in the Anglo-sample dataset). Clinicians described supporting participants in recognizing signs of overstimulation (the Anglo-sample dataset mentions understimulation as well), tracking time spent in virtual environments, and identifying when to pause or disengage in order to regulate activation. Structured breaks and access to spaces for recovery were framed as essential components to avoid prolonged immersion in virtual contexts and to support te-engagement with offline reality.

By contrast, international clinicians emphasized neurotype composition as the key to intervention facilitation. They explain how who is in the social space can influence how they feel they are being perceived or are perceived and this shapes behavior, suggesting that autistic-dominant or neurodivergent spaces may inherently reduce threat and allow for natural social autistic expression and socialization:

“Having other people with your neurotyping…. THAT changes the whole dynamic” (Interview 1, USA)

In this perspective, who is present in the interpersonal space — and how one is perceived — shapes social behavior more strongly than external structuring.

Overall, Italian clinicians tended to locate safety in relational and environmental structure and control mediated by professional presence, whereas international clinicians emphasized relational symmetry and neurotype alignment as intrinsic sources of safety. These positions reflect different, yet potentially complementary, design logics for socio-relational interventions.

##### 3.3.2.4 Divergence 4: intersectionality as a framework vs. a contextual consideration

International clinicians spontaneously integrated intersectionality (i.e., race, class, gender, LGBTQIA + identity) into what “support” must account for, including how privilege shapes available choices and safety. While inclusivity is present in the Italian sample, the international interviews treated intersectionality as a primary organizing frame rather than a contextual consideration.

This divergence has identifiable roots in the different institutional and advocacy contexts from which the two samples were drawn. In the United States and Australia, the integration of intersectionality into clinical and therapeutic training has been significantly shaped by disability justice movements that explicitly link autism, race, gender, and sexuality as overlapping axes of structural disadvantages [e.g., (71)–(75)]. The concept of “autistic LGBTQIA + identity” has received increasing attention in research and practice in these contexts, reflecting findings that autistic populations show higher rates of gender diversity and non-heterosexual orientation than the general population [i.e., (76, 77)], and that this intersection carries distinct vulnerabilities and support needs. The international clinicians’ emphasis on intersectionality is therefore consistent with a broader cultural shift in English-language clinical psychology and social work toward justice-oriented practice frameworks.

In Italy, clinical understandings and approaches to autism have been primarily shaped by neuropsychiatric and neurobiological traditions [“medicalization of autism”; (51)], reflecting a historically medicalized system organized around diagnostic categories and standardized clinical protocols. Against this backdrop, sociopolitical and identity-based frameworks that are more established internationally (i.e., neurodiversity, intersectionality, and strengths-based paradigms) have only recently begun to circulate within Italian autism discourse, where their uptake remains uneven and is marked by polarisation between proponents, sceptics, and those who reject these frameworks outright (52). While intersectionality is present in Italian scholarship [e.g., (78)], it appears less systematically formalized within mainstream cynical discourse and service organization, though some practitioners may informally integrate it at an individual level. This does not necessarily signify there is an absence of awareness, but rather it reflects differences in how structural and political dimensions of or concerns regarding disability are incorporated into clinical epistemologies and practice. Future research engaging Italian clinicians specifically on intersectionality — and on the support needs of autistic people with intersecting marginalized identities — would be valuable in clarifying whether this divergence also reflects a genuine gap in provision or differences in how these concerns are articulated.

##### 3.3.2.5 Divergence 5: salient risk focus: avoidance and overuse vs. relational ambiguity and rejection

Italian clinicians most often highlighted the danger of SVEs becoming an avoidant refuge:

“Positioned between social withdrawal and in-person interaction, virtual reality may represent an intermediate option; however, it also carries the risk of functioning as avoidance” (FG7)

International clinicians emphasized relational ambiguity (e.g., ghosting, mixed signals) as a specific vulnerability for autistic adults:

“Autistic people assume what you say is what you mean… and then there's no resolution” (Interview 2, USA)

These risks operate at different levels: Italian clinicians focus on behavioral withdrawal, international clinicians on emotional injury. Both are clinically significant and should be addressed in intervention design and implementation.

Taken together, the Italian and international datasets converge on the view that social virtual environments may offer meaningful opportunities for connection and engagement for autistic young adults, provided that participation is structured, personalized, and safety-oriented. Divergences between datasets highlight culturally distinct emphases in how support is conceptualized — either through structured competence- or skill-building or through identity-affirming agency — suggesting that effective SVE-based interventions may need to integrate both logics rather than privilege one exclusively.

## 4 Discussion

This study contributes clinician-informed insight into what it would mean to develop socio-relational interventions for autistic young adults within social virtual environments (SVEs). Rather than treating SVEs as inherently therapeutic, clinicians tended to frame them as social ecologies whose effects depend on conditions — including how safety is produced, how autonomy is protected, and how relational risk is anticipated. Below, we interpret these findings through three analytic lenses that extend beyond description: (i) SVEs as “conditional social infrastructures”, (ii) two complementary logics of support, and (iii) trauma-informed ethics as an emergent cross-context concern. We then situate these lenses within relevant theoretical frameworks and discuss implications for how neuro-affirming practice should be supported in SVE contexts from a clinician's perspective.

### 4.1 SVEs as “conditional social infrastructures”, not therapeutic technologies

A key interpretive implication is that clinicians did not primarily evaluate SVEs as “technologies”, but as contexts for social participation. This distinction may be relevant for intervention science. It aligns with the broader shift in Extended Reality (XR) research away from novelty and toward appropriating existing tools for specific populations and goals (27; 28), while foregrounding the ecological conditions under which technology becomes supportive rather than harmful. In this framing, the question becomes less “does VR work and can it deliver effective interventions?” and more “what must be true about the environment — socially, sensorially, relationally — for participation to be sustainable?”.

Conceptualizing SVEs as conditional infrastructures may also help explain why clinicians repeatedly focus on boundaries and integration with offline life: the clinical object is not immersion itself, but the management of access, demand, and transfer across settings.

This also repositions “risk” from being a property of autistic people (e.g., presumed vulnerability) to being a property of misfit between people and environments, consistent with neurodiversity-affirming and ecological perspectives. That shift is potentially useful: it directs future trials toward testable moderators (sensory controllability, group composition, facilitation stance, pacing) rather than toward static participant deficits.

### 4.2 Two support logics that can be productively integrated

Cross-context differences in clinicians’ emphasis can be interpreted as two support logics that are not mutually exclusive but reflect different starting points about what creates change.

One logic prioritizes structured scaffolding and competence-building. Under this view, autonomy is enabled through predictability, mediated interaction, gradual exposure, and deliberate generalization beyond the therapeutic setting.

The second logic prioritizes identity-affirming agency, suggesting that adult support should expand choice instead of enforcing compliance with normative social expectations. From this perspective, clinicians treat authenticity, masking, and social participation as ethically conditioned by safety and power, not as moral imperatives. Instead of being tangential, identity-affirming practices (e.g., language preference, relational symmetry, intersectional awareness) can be understood as contributing to the conditions of safety and participation in social environments, shaping how autistic individuals engage, regulate, and sustain social interaction. In this sense, they appear analytically relevant to the design of SVE-based interventions (rather than being reducible to peripheral ethical considerations) and in other autism-informed interventions.

Importantly, these logics can be understood as addressing different failure modes of adult support. Over-structuring can drift into normalization-by-stealth; under-structuring can drift into abandonment-by-design (placing the burden of navigation and harm management on the autistic person). The interpretive contribution of this study is the suggestion that effective SVE interventions may benefit from a hybrid architecture: scaffolding that is explicitly offered as a resource (not a demand), guided by an ethical commitment to autonomy and identity integration. This integration offers a way to bridge the “skill vs. acceptance” polarization that has historically shaped autism interventions and to reframe intervention success as sustainable participation on one's own terms.

### 4.3 Trauma-informed ethics

A further interpretive insight is that international clinicians’ accounts frequently reflected elements of trauma-informed reasoning (79)–(81) even when not explicitly labeled as such. Their focus on emotional safety, relational rupture, rejection sensitivity, and the pragmatic necessity of masking indicates a clinical epistemology attentive to cumulative relational harm and power asymmetries [i.e., literature on autistic polyvictimization in adulthood, example: (38)]. This appears particularly relevant to SVEs, which may simultaneously reduce certain barriers to interaction and heighten exposure to ambiguous, unstable, or abruptly terminating relationships. In other words, SVEs may lower entry costs while potentially increasing exposure to specific relational risks and/or injuries (e.g., ghosting, unclear expectations).

This suggests that SVE interventions, if developed, may need to move beyond simply transplanting social skills curricula into virtual spaces. They may benefit from incorporating trauma-informed elements — anticipatory psychoeducation about relational ambiguity, explicit repair scripts, and safety planning — while avoiding the pathologization of protective adaptations. In addition, with the Italian clinicians’ emphasis on containment and boundedness, the combined dataset may suggest that “trauma-informed” and “scaffolding” approaches are not competing philosophies but mutually reinforcing strategies for harm reduction.

### 4.4 Theoretical positioning: double empathy problem, third places, and epistemic justice

Several theoretical lines can be brought into dialogue with these interpretations. First, clinicians’ attention to neurotype composition and “being perceived” is consistent with the Double Empathy Problem framework: social breakdown is bidirectional and context-dependent, not located solely within autistic individuals (14). Second, clinicians’ focus on belonging and community access resonates with “Third Place Theory” conceptualizations (29) — spaces outside home and work where affiliation and identity can develop through low-stakes participation. SVEs may function as such spaces for some autistic adults, but only if they are designed to avoid substitution, exclusion, or unmoderated harm.

Finally, the findings align with epistemic justice critiques in autism-related fields [e.g., (82)]: support must not be designed around neurotypical comfort as the unspoken endpoint. A discussion of SVEs as interventions therefore necessarily includes ethical questions about whose norms are centered, whose burden is reduced, and who holds interpretive authority in deciding what counts as improvement.

### 4.5 Clinicians’ neurotype: as an inherent bias or as an interpretive point of view?

The cross-continental differences observed likely reflect more than just national culture differences; they are also plausibly shaped by the clinicians’ own neurotype-standpoints. The international sample (entirely neurodivergent) may have amplified attention to environmental and relational harm as lived, or embodied, realities; in fact, the Angloclinicians shared personal anecdotal examples of autistic lived experience in ways that shaped their clinical framing. The Italian sample may have fostered a stronger emphasis on structured mediation and generalization through traditional, conventional clinical reasoning because predominantly neurotypical/allistic, to our knowledge.

We propose that this should not be interpreted as a “bias” to be eliminated but as an analytic signal: different forms of expertise might not just be valuable but necessary to have, in order to design interventions that are effective in ways that account for (1) external perspectives and social expectations surrounding autism interventions, and (2) internally experienced dimensions of autism and intervention use. This distinction (clinical expertise grounded solely in professional work with autistic individuals vs. clinical expertise that integrates professional knowledge with lived autistic experience) has implications for the composition of future co-design teams, which would ideally include autistic clinicians, autistic end-users, and non-autistic clinicians in complementary roles.

### 4.6 Limitations and future directions

A limitation of this study is the relatively small international (Anglo) sample (*N* = 5), which does not allow for broad generalization across U.S./Australian clinical contexts (even though data sufficiency was reached for the analytic purpose of comparison). However, these interviews were included as a comparative analytic extension rather than as a representative sample, and they provided conceptually rich insights that supported the identification of cross-cultural convergences and divergences. Accordingly, international findings are presented as contextually situated perspectives (insights) and hypothesis-generating observations, and not as exhaustive claims. Moreover, the findings are grounded in clinician perspectives rather than autistic young adults’ direct accounts; clinicians can anticipate feasibility and ethical risk, but participatory design with autistic end-users is essential for legitimacy and ecological validity. The study also focuses on autistic adults with low support needs, limiting transferability to people with higher support needs or different communication profiles. Future research should extend this work to *(a)* autistic young adults with diversified profiles and *(b)* through larger and more diverse cross-cultural samples (e.g., autistic clinicians from additional, different nationalities and from settings serving autistic people with a broader range of support needs and communication profiles).

Another limitation of the present study is that it focuses on clinicians’ perspectives rather than directly analyzing autistic adults’ own accounts of SVE-based support. Although the international clinician sample included neurodivergent professionals, primarily autistic and/or ADHD, this does not eliminate the need for direct end-user, autistic adult participation. Neurodivergen clinicians may offer an important hybrid standpoint, combining professional expertise with live neurodivergent experience, but their perspectives remain shaped by clinical roles, professional responsibilities, and service-design concerns. Future research should therefore extend this work through participatory and co-design approaches that foreground autistic adults as primary knowledge-holders in defining what counts as safety, accessibility, belonging, autonomy, and meaningful social participation in SVEs.

Finally, the study does not evaluate an implemented SVE intervention; it delineates clinically informed insights about mechanisms and risks that now require empirical testing. Future research should therefore prioritize: (i) participatory co-design with autistic adults, (ii) pilot studies with explicit safety protocols and measurements of belonging, loneliness and harm indicators, and (iii) mechanism-focused evaluation (e.g., whether neurotype composition, facilitation stance, and sensory controllability predict engagement and outcomes). Cross-context/-cultural work should also examine how ethical priorities shift across systems of care and how those shifts shape implementation.

### 4.7 Implications for SVE interventions

In practical terms, the findings suggest that SVE interventions may be most effective when designed as conditional infrastructures: scaffolded, safety-oriented, and autonomy-preserving. The findings do not suggest that SVEs should replace in-person social participation, but that they may offer a regulated intermediate ecology for engagement, connection, and skill transfer — especially when conventional in-person settings are inaccessible or excessively demanding (e.g., joining peers at a club In-Real-Life *vs.* in a controlled virtual environment where social exposure, sensory stimuli, etc. can be personalized and adjusted according to individual preferences and needs).

Clinicians seeking to support autistic young adults in SVEs should consider embracing a neuro-affirming approach. The findings point to several concrete priorities: establishing psychologically safe group compositions (i.e., including consideration of neurotype); providing explicit preparatory scaffolding (i.e., structured orientation to virtual platform features and affordances); ensuring professional mediation during session; embedding build-in pause and exit options; providing psychoeducation about relational dynamics specific to online environments (i.e., the possibility of ghosting instances); and framing all support activities as resources rather than demands.

Future intervention development should aim to integrate both support logics identified (structured competence-building and identity-affirming agency), while paying attention to the cultural assumptions that shape expectations surrounding autonomy, guidance, communication, and social participation. Cross-cultural scholarship has long emphasized that meanings attached to interpersonal behavior and support practices are not universal but culturally mediated [e.g., (83, 84)]. Interventions should therefore embed trauma-informed safeguards while maintaining flexibility toward culturally variable understandings of relationality, independence, and care, to reduce foreseeable harms.

## 5 Conclusions

The divergences identified in how professionals conceptualize “support” represent more than a difference in emphasis; they reflect a conceptual tension that invites reconsideration of the very definition and operationalization of “*intervention*” in autism research and practice. Whereas the Italian perspective tends to frame intervention as a process of structured guidance and adaptation, the Anglo perspective positions it as a pathway toward identity affirmation. Our findings suggest that interventions designed for SVEs may not be fully captured by approaches that treat them solely as environments for skill acquisition or the removal of barriers. Instead, they may be more appropriately reconceptualized as negotiated spaces that balance support, autonomy, and identity integration — spaces that are sensitive to cultural context while remaining responsive to the lived experiences and realities of autistic adults. These conceptual tensions are not unique to virtual environments; they also echo broader unresolved questions in autism intervention science about what counts as “improvement”, whose standards apply, and who holds interpretive authority [i.e., (85)–(89)]. Addressing these questions in the specific context of SVEs may offer productive ground for developing the next generation of autistic-affirming, empirically grounded interventions.

As a future direction, we plan to gather insights directly from autistic young adults and subsequently pilot test and evaluate a social VR intervention design developed from this and the forthcoming participatory study.

## Data Availability

Anonymized transcripts, coded excerpts and/or other analytic materials are available upon reasonable request to the corresponding author, subject to ethical review and data use agreements. Requests to access the datasets should be directed to paola.venuti@unitn.it.
